# Osteology of the first skull of *Aetosauroides scagliai* Casamiquela 1960 (Archosauria: Aetosauria) from the Upper Triassic of southern Brazil (*Hyperodapedon* Assemblage Zone) and its phylogenetic importance

**DOI:** 10.1371/journal.pone.0201450

**Published:** 2018-08-15

**Authors:** Ana Carolina Biacchi Brust, Julia Brenda Desojo, Cesar Leandro Schultz, Voltaire Dutra Paes-Neto, Átila Augusto Stock Da-Rosa

**Affiliations:** 1 Programa de Pós-Graduação em Geociências, Instituto de Geociências, Universidade Federal do Rio Grande do Sul, Porto Alegre, RS, Brazil; 2 División Paleontología Vertebrados, Museo de La Plata, Facultad de Ciencias Naturales y Museo, La Plata, Buenos Aires, Argentina; 3 Departamento de Paleontologia e Estratigrafia, Instituto de Geociências, Universidade Federal do Rio Grande do Sul, Porto Alegre, RS, Brazil; 4 Departamento de Geociências, Centro de Ciências Naturais e Exatas, Universidade Federal de Santa Maria, Santa Maria, RS, Brazil; Royal Belgian Institute of Natural Sciences, BELGIUM

## Abstract

Aetosauria, which includes 30 species, is a diverse group of armored pseudosuchian archosaurs restricted to Upper Triassic beds. Three species occur in Brazil, and one of these, *Aetosauroides scagliai* Casamiquela, 1960, also occurs in Argentina. The specimen UFSM 11505, found at Faixa Nova–Cerrito I Outcrop, Santa Maria Formation (*Hyperodapedon* Assemblage Zone), Santa Maria, Rio Grande do Sul State, Brazil, is here referred to as *Aetosauroides scagliai*. This specimen preserves most of the skull with both hemimandibles in association with most of the postcranium, thus representing one of the most complete aetosaur skeletons found in Brazil. The premaxilla, one of the key elements of the cranial morphology of aetosaurs, along with the posterior portion of the mandible, was not described until now for *A*. *scagliai*. In contrast to the typothoracinae aetosaurs, the premaxilla of UFSM 11505 presents a shovel-shaped tip, but it is not as prominent as the lateral expansion of desmatosuchian aetosaurs, including both species of *Stagonolepis*, *S*. *robertsoni* Agassiz, 1844 and *S*. *olenkae* Sulej, 2010. The retroarticular process of the mandible is elongate and not tall, as in *Stenomity huangae* Small & Martz, 2013 and other typothoracinae aetosaurs. Unlike previous descriptions of *A*. *scagliai*, the maxillary teeth are recurved ziphodont-like with serrations on the entire length of both margins. Premaxillary teeth are also present, being less recurved than the maxillary teeth and cylindrical. We recovered *Aetosauroides scagliai* as the most basal taxon within Aetosauria, like previous phylogenetic analyses. Furthermore, our analyses reinforce that recurved and unconstricted maxillary teeth, the shovel-shaped premaxilla and the presence of a tuber on the surangular are plesiomorphic features of Aetosauria.

## Introduction

Aetosauria is a group of quadrupedal pseudosuchian archosaurs, covered by dorsal, ventral, and appendicular osteoderms, and restricted to the Upper Triassic [1]. The first reported materials were osteoderms found in the upper layers of the Old Red Sandstone, Scotland, and mistakenly described as glenoid scales of *Stagonolepis robertsoni*, at the time considered a sarcopterygian fish [2]. Huxley [3] reconsidered *S*. *robertsoni* to be a crocodilian reptile. However, aetosaurs were only recognized in 1877, when Oscar Friedrich von Fraas described *Aetosaurus ferratus* based on a block containing 24 articulated skeletons, found at the village of Kaltental, Germany [4]. Later, Nicholson & Lydekker [5] defined “Aetosauria” to include *A*. *ferratus* [4] and *Typothorax coccinarum* [6], from the Chinle Formation, New Mexico, USA. *S*. *robertsoni*, in turn, would first be recognized as an aetosaur in 1961 [7]. In subsequent years, more specimens were found worldwide and assigned to this group, including 30 species today. The diagnostic osteoderm morphology of aetosaurs allowed other authors to diagnose several taxa, and propose a biostratigraphic application as an index for Upper Triassic continental strata [1, 8–14]. However, there are similar patterns of ornamentation expressed and shared in several species, such as *Aetosauroides scagliai* [15], *Neoaetosauroides engaeus* [16], *Coahomasuchus kahleorum* [17] and *Stenomyti huangae* [18], as discussed by some authors the last ten years [1, 19, 20]. Therefore, isolated osteoderms would not be sufficient to distinguish species.

In South America, five species are known: *A*. *scagliai*; *N*. *engaeus*; *Chilenosuchus forttae* [21]; *Aetobarbakinoides brasiliensis* [22]; and *Polesinesuchus aurelioi* [23]. Three of these occur in Brazil, *A*. *brasiliensis*, *P*. *aurelioi* and *A*. *scagliai*, the latter being the only one also recorded in Argentina [24]. Most of them are represented by osteoderms and postcranial material, whereas *N*. *engaeus* is the only South American aetosaur known from several skulls [25–27].

Casamiquela first described *A*. *scagliai* in 1960 [15] and shortly afterwards improved upon its description based on other specimens, PVL 2073, PVL 2059, PVL 2014 and PVL 2052, all of them from the Ischigualasto Formation, Argentina, and constitute most of the axial and appendicular skeleton as well as articulated osteoderms [15, 28, 29].

The taxonomic history of *A*. *scalgiai* in Brazil is complex. In 1982, Zacarias [30] informally described a new *Aetosauroides* species, “*A*. *subsulcatus*” in her unpublished master’s thesis, based on material from the Santa Maria Formation, Brazil. Three years later, Barberena *et al*. [31] changed its name to “*A*. *inhamandensis*” with no written justification. Later, Heckert & Lucas [11] synonymized *A*. *scagliai* with *Stagonolepis robertsoni* [2], based mostly on post-cranial character states. Both *“A*. *subsulcatus”* and “*A*. *inhamandensis*” in subsequent studies were considered *nomina nuda* and then synonymized with *A*. *scagliai*, along with the description of apomorphies, which distinguished *A*. *scagliai* from *S*. *robertsoni* [24].

Although known by fairly complete material, most of the skull of *A*. *scagliai* is still unknown, including the premaxilla and its teeth. As *A*. *scagliai* is recovered as the most basal and sister-taxon of all other members of Aetosauria [22, 32, 33], it is important to identify and clarify yet unknown cranial character states. In this contribution, we describe new skull material of *Aetosauroides scagliai* from the Santa Maria Supersequence, Brazil, and for the first time includes the anterior portion of the rostrum, which allows a more complete reconstruction of its skull.

### SYSTEMATIC PALEONTOLOGY

ARCHOSAURIA Cope, 1869 [34] *sensu* Gauthier & Padian, 1985 [35]

PSEUDOSUCHIA Zittel, 1887–1890 [36] *sensu* Gauthier, 1985 [35]

AETOSAURIA Marsh, 1884 [37] *sensu* Parker, 2007 [19]

*AETOSAUROIDES SCAGLIAI* Casamiquela, 1960 [15]

= “*Aetosauroides subsulcatus”* Zacarias, 1982 [30]

= “*Aetosauroides inhamandensis”* Barberena *et al*., 1985 [31]

= *Argentinosuchus bonapartei* Desojo, 2005 [38]; Ezcurra, 2016 [39]

Type species: *Aetosauroides scagliai* Casamiquela, 1960 [15]

Diagnosis: As for type species.

Holotype: PVL 2073, incomplete and articulated postcranial skeleton: one anterior cervical centrum (2073–40), 13 dorsal, two sacral, and seven caudal vertebrae (2073–11), eight incomplete ribs, including two proximal ends articulated to their corresponding anterior dorsal vertebrae, almost complete right scapula (2073–14), articulated proximal portion of left scapula, coracoid, interclavicle, and clavicle (2073–15), incomplete right humerus (2073–6), complete left humerus (2073–3), ulna (2073–5), radius (2073–4), a pair of metacarpals (2073–33/34), articulated and complete ilia (2073–11), pubes lacking their distal ends (2073–17/18), almost complete and articulated ischia (2073–16), complete right femur (2073–2), fibula, astragalus, and distal tarsal (2073–31), complete left femur (2073–1), tibia lacking its distal end (2073–7), complete fibula (2073–8), probable proximal tarsal (2073–30), two metatarsals lacking their distal ends (2073–9/10), two isolated metatarsals (2073–19/20), pedal non-terminal phalanges (2073–35/37), an ungual (2073–32), paramedian and left lateral osteoderms of the cervical, dorsal, sacral, and caudal regions (2073–38), ventral osteoderms (2073–22), several appendicular osteoderms (2073–21/43), fragments of indeterminate osteoderms (2073–39/40), and some indeterminate fragments of bone (2073–53) [24].

Horizon and locality: Cancha de Bochas Member, Ischigualasto Formation, Argentina [40].

Stratigraphic range: Upper Triassic (Carnian) [41].

Revised diagnosis: Small to medium-sized aetosaur (1 to 2.42 meter in length) distinguished from other aetosaurs by the following apomorphies (autapomorphies with asterisk): maxilla excluded from the margin of the external naris*; ventral margin of dentary convex and without a sharp inflexion*; dorsal margin of the surangular with presence of a rounded tuber; recurved tooth crowns with denticles (ca. 8 per mm) on both mesial and distal margins without either wear facets or constriction between root and crown*; cervical and dorsal centra with oval fossae ventral to the neurocentral suture on the lateral sides of the centra; mid- and posterior dorsals with well-developed posterior infradiapophyseal lamina directly ventral to the diapophyses, and postzygapophyses posterolaterally divergent; ratio between the entire length of the postzygapophyses and the width between the posterior-most tips of the postzygapophyses equal or lower than 0.75*; anterior tip of premaxilla slightly expanded laterally (incipient shovel-shaped), contrasting with the well laterally expanded anterior tip of *Stagonolepis* and *Desmatosuchus* (see Discussion).

Referred material: (1) PVL 2052, a large-sized specimen with some skull elements preserved as natural casts, with much of the posterior portion of the postcranial skeleton well preserved, including the posterior dorsal vertebrae, pelvic girdle, fragments of the limb-bones and several articulated and isolated dorsal paramedian osteoderms including articulated tail armor [29]; (2) PVL 2059, small to medium-sized specimen with a partially preserved skull, with the anterior portion of the carapace preserved in articulation associated with correspondent region of the axial skeleton [15, 28]; (3) MCP-13, a small-sized specimen represented by six articulated dorsal vertebrae, partial articulated dorsal and ventral armor, several isolated lateral and ventral osteoderms, and fragments of vertebrae, ribs and osteoderms [43]; (4) UFSM 11070 (= MCP 3450 and UFRGS-PV-1302-T) a small to medium-sized specimen with most of the posterior portion of the postcranium [24, 44]; (5) UFSM 11505, skull with both hemimandibles, associated postcranium, including dorsal and caudal vertebrae, right pubis, an almost complete right hind-limb, and dorsal trunk paramedian osteoderms and ventral osteoderms (see below).

**Fig 1:**
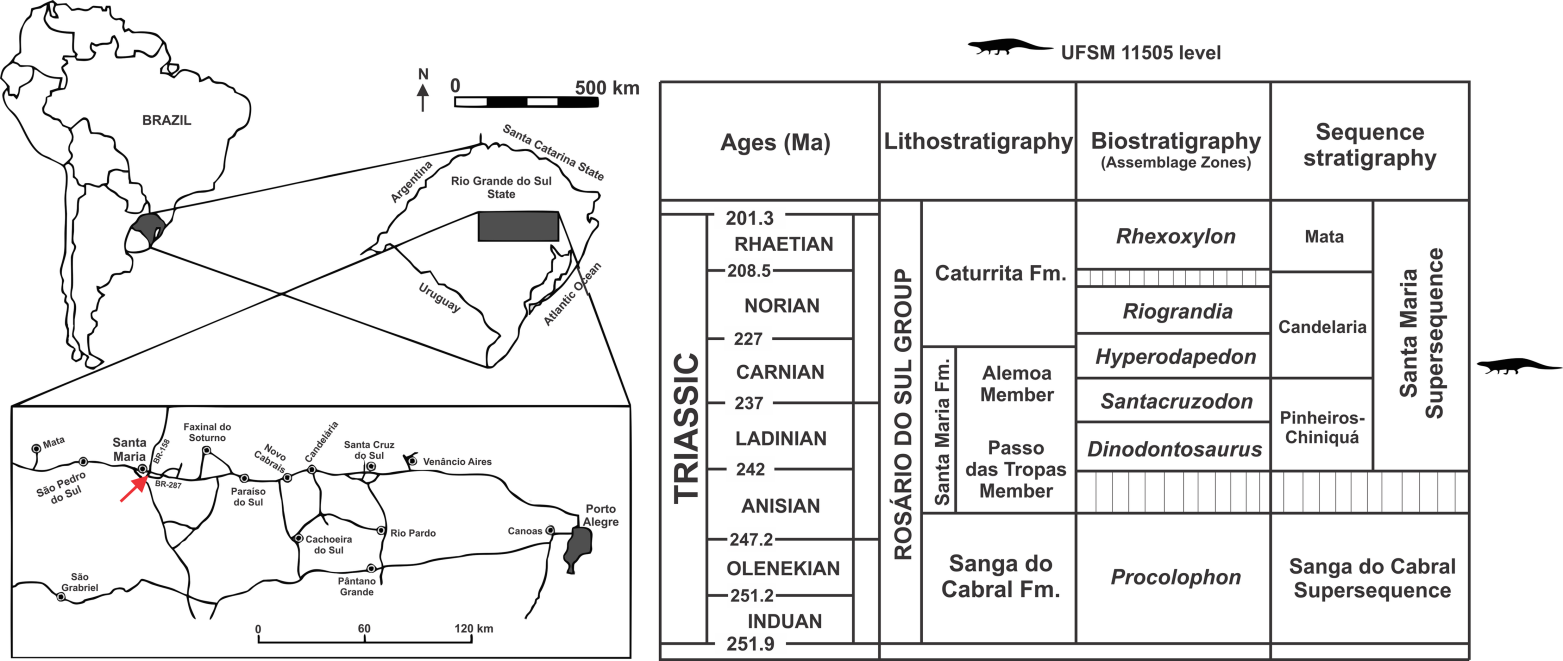
Locality and horizon of UFSM 11505. Map of the Brazilian region showing the Faixa Nova–Cerrito I Outcrop (arrow) in the Santa Maria Formation (modified from Desojo *et al*. [22]) and chronostratigraphic diagram for the Triassic of southern Brazil (modified from Da-Rosa [47]). Ages according to Cohen *et al*. [36], and sequence stratigraphy according to Horn *et al*. [45].

UFSM 11505 horizon and locality: massive red beds from the Santa Maria Formation, as part of the Candelária Sequence, Santa Maria Supersequence, Rio Grande do Sul, Brazil [45], found at the Faixa Nova–Cerrito I outcrop [46, 47] (Fig 1).

## Materials & methods

The material studied here is registered under the number UFSM 11505 and hosted at the fossil collection of the Laboratório de Estratigrafia e Paleobiologia of Universidade Federal de Santa Maria (UFSM), Santa Maria, Rio Grande do Sul State, Brazil. No permits were required for the described study according to Brazilian's Federative Constitution of 1988 and Law 11.738/02 of December 13th of 2001 of Rio Grande do Sul State, Brazil.

**Fig 2:**
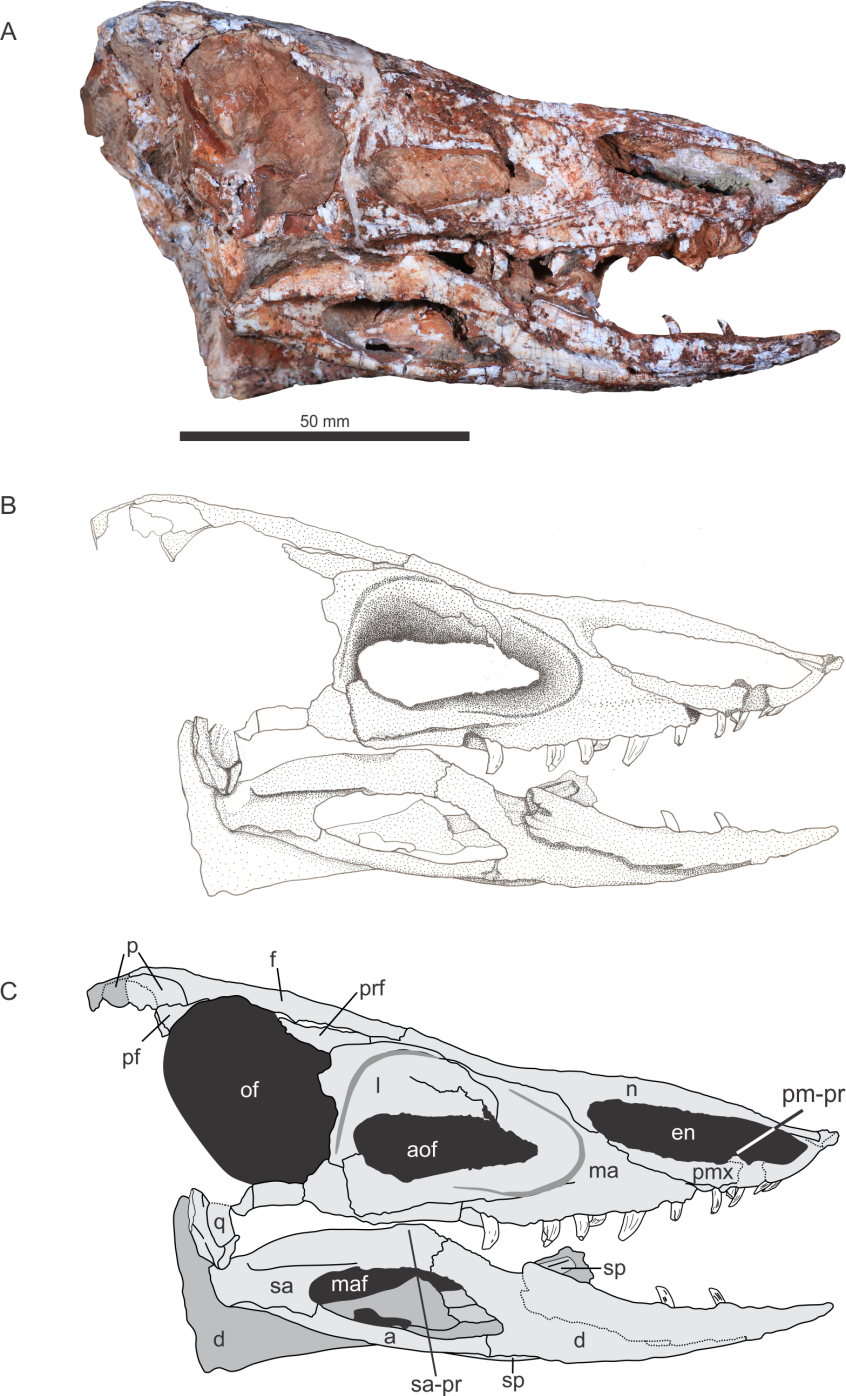
Skull of referred specimen of *Aetosauroides scagliai* (UFSM 11505), right lateral view. Abbreviations: a, angular; aof, antorbital fenestra; d, dentary; en, external naris; f, frontal; l, lacrimal; ma, maxilla; maf, mandibular fenestra; n, nasal; of, orbital fenestra; p, parietal; pf, postfrontal; pm-pr, dorsal projection of premaxilla; pmx, premaxilla; prf, prefrontal; q, quadrate; sa, surangular; sa-pr, dorsal tuber of surangular; sp, splenial.

**Fig 3:**
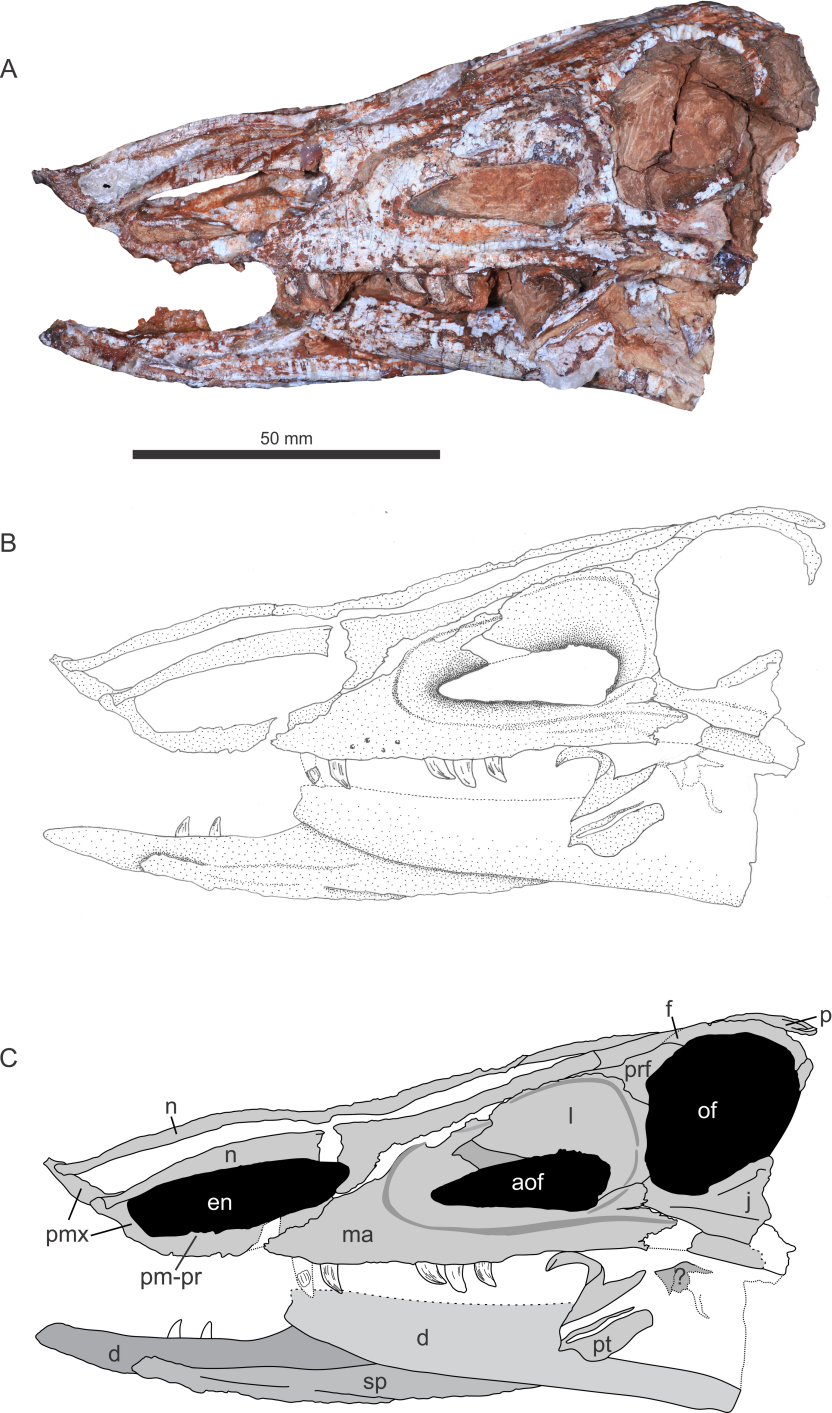
Skull of referred specimen of *Aetosauroides scagliai* (UFSM 11505), left lateral view. Abbreviations: same as Fig 2.

**Fig 4:**
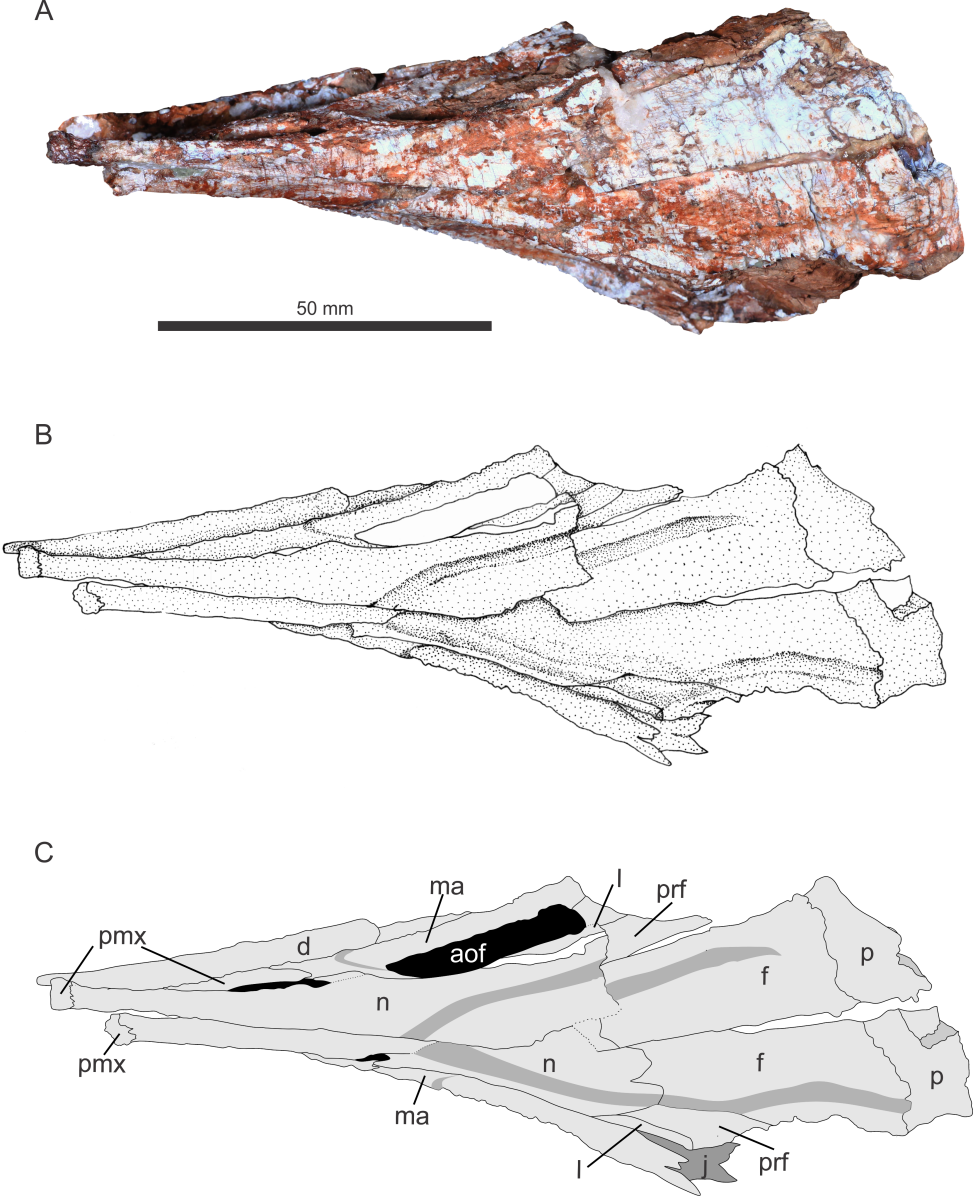
Skull of referred specimen of *Aetosauroides scagliai* (UFSM 11505), dorsal view. Abbreviations: same as Fig 2.

The specimen was found in 2009 by a team of researchers of the Universidade Federal de Santa Maria and Universidade Federal do Oeste do Paraná at Faixa Nova–Cerrito I Outcrop [44], at the meeting point between BR-287 and BR-158 roads, in the city of Santa Maria, Rio Grande do Sul State, Brazil (Figs 2–4). This outcrop is characterized by medium to fine-grained mudstones typical of the base of the Candelária Sequence (Upper Triassic, Carnian), one of the four third-order sequences of the Santa Maria Supersequence [45, 48, 49] (Fig 1). Several specimens of the rhynchosaur *Hyperodapedon* were also previously collected from this outcrop (UFRGS-PV-0408-T and UFRGS-1302-T, personally identified by VDPN and CLS), linking Faixa Nova–Cerrito I Outcrop to the *Hyperodapedon* Assemblage Zone [24, 50]. UFSM 11505 was divided in the field into several blocks to be moved and, unfortunately, the skull was broken in half and had to be reassembled.

UFSM 11505 consists of a skull with both hemimandibles (Figs 2–4) andassociated postcranial material. Here we concentrated on the cranial description because it preserves unknown elements for the species. Unfortunately, parts of postcranial material are yet unprepared and we used available postcranial information based on the description of [51] for the diagnosis and phylogenetic analysis only. All of the postcranial information will be used in a future study focusing on the biomechanics of the species.

## Results

### General remarks

The specimen is referred to *Aetosauroides scagliai* based on the following character states shared between UFSM 11505 and the holotype of *A*. *scagliai*: maxilla excluded from the margin of the external naris (see discussion); ventral margin of dentary convex and without sharp inflexion; tooth crowns with straight distal margins and without constriction between root and crown, denticles (see discussion), and no wear facets on the teeth; dorsal centra with oval fossae ventral to the neurocentral suture on the lateral sides of the centra; mid dorsals with well-developed posterior infradiapophyseal lamina directly below the diapophyses, and postzygapophyses posterolaterally divergent, ratio between the entire length of the postzygapophyses and the width between the distal-most tips of the postzygapophyses equal or lower than 0.75 [24].

Desojo & Ezcurra [24] described a lack of denticles on tooth crowns of *A*. *scagliai*. However, one of the authors, J. B. Desojo, noted them on the dentary teeth of PVL 2059 after a more detailed examination. Denticles were also observed in UFSM 11505 and are herein described. The tooth description is based on the proposed terminology by Hendrickx *et al*. [52].

Based on the presence of closed and partially closed neurocentral sutures on the available dorsal and caudal vertebrae [53, 54], UFSM 11505 is more skeletally mature than MCP-13 and PVL 2070, both *A*. *scagliai* specimens with an estimated age of 2 and 5 years old respectively [43, 55]. UFSM 11505 is probably a mature specimen based on its estimated size (1.45 meters in length) in comparison with the one-meter long MCP-13 [43] and the holotype (PVL 2073: 1.39 meters estimated length) [43].

Most of the cranial sutures in UFSM 11505 are unfused. Notwithstanding, according to Bailleul & Horner [56], cranial sutures may not fuse progressively during ontogeny in all archosaurs, being able to become even wider. Therefore, the presence of unfused cranial sutures does not imply that UFSM 11505 was an immature individual. Since paleohistological analyses are under way, it is not possible yet to estimate the age of UFSM 11505.

### Description of UFSM 11505

The skull is medio-laterally compressed. The right side of the skull was displaced forward, the mid-anterior left side displaced towards the broken mid-anterior right side, so the left hemimandible slid towards the midline, ending up between the maxilla and the right hemimandible. In dorsal view, there is a rupture situated diagonal to the midline beginning from the suture between the right frontal and the right nasal to the medial portion at the left nasal, and separating the skull from side to side. This fracture was repaired and both parts were glued together during preparation.

The posterior part of the skull preserves the occiput, but the braincase is lost. However, it is still possible to observe some elements of the posterior region which were displaced, e.g. quadrate, and elements of the palatal complex, e.g. pterygoids. The latter are displaced, on the left side, between the jugal and the posterior part of the left mandible. Measurements of elements are in Table 1.

**Table 1:** Maximum measurements (in mm) of the individual elements, fenestrae and fossae of the skull of UFSM 11505. Asterisk: incomplete; dash: not preserved.

	RIGHT		LEFT	
	Length	Height	Length	Height
External naris	39	9	39,8	11
Antorbital fenestra	32,9	12	32,3	10,8
Antorbital fossa	42,2	24	45,8	25,5
Orbital fenestra	28,8	33	28,5	32
Mandibular fenestra	37,2	13,8	-	-
	Width	Length	Width	Length
Nasal	12,5	81,5	11	82
Frontal	16	40	16,7	38,5
Parietal	22	?	20,5*	?
Lacrimal	30,45	20	21,5	31,2
Dentary	15	72,8	*	74,9*
Angular	4,8	52,1	-	-
Surangular	12,1	42	-	-
	WIDTH		LENGTH	
TOTAL	44		136*	

The teeth are well preserved. Maxillary teeth are present on both sides, albeit incomplete, and all premaxillary teeth are present only on the right side.

#### Premaxilla

Both premaxillae are well preserved. Each one is very narrow compared to the maximum width of the skull. The anterior and posterodorsal processes encircle the external naris as in other aetosaurs (Figs 2–4). The posterodorsal process contacts and is overlapped by the anterior process of the nasal. The anterior process of the premaxilla continues anteriorly to form, in lateral view, an anteroposteriorly expanded tip to the contact between the nasal and the premaxilla. In dorsal view, the anterior tip of the premaxilla slightly expands laterally, forming a weakly developed shovel-shape, contrasting with the well-developed shovel-shaped expansion of *Stagonolepis robertsoni* [7] and *Desmatosuchus smalli* [42, 57]. This conditioncontrasts with the snout of *Aetosaurus ferratus* [58], *Paratypothorax andressorum* [32] and *Stenomyti huangae* [18] which all lack a lateral expansion. The posterior process of the premaxilla outlines the ventral margin of the external naris. Although broken, it contacts the ventral process of the nasal and excludes the maxilla from the margin of the external naris, as in PVL 2073 and UFSM 11505, respectively the holotype and referred specimens of *Aetosauroides scagliai*.On the dorsal surface of the posterior process of the premaxilla, within the external naris, a small tubercle is present at the level of the third premaxillary tooth. This tubercle is present in several species of aetosaurs, such as *Stagonolepis olenkae* [59], *Paratypothorax andressorum* [32] and *Desmatosuchus smallii* [42, 57], although being taller in these species than in UFSM 11505. The right premaxilla is broken and it bears, in its mid-posterior portion, five slender teeth. These teeth are smaller in size than the maxillary teeth. The left premaxilla teeth have been broken, and therefore only three alveoli are seen. The anteriormost portion of the premaxilla is edentulous, as in *A*. *ferratus* [58], *P*. *andressorum* [32] and *S*. *robertsoni* [7].

#### Maxilla

The maxilla is the most anteroposteriorly extensive bone in the lateral view of the skull, anteriorly extending to the mid-point of the external naris, posteriorly to the posterior end of the antorbital fenestra and dorsally bounds the anterior part of the antorbital fenestra (Figs 2 and 3). There is a well-marked antorbital fossa surrounding the antorbital fenestra, as in *Stagonolepis olenkae* [59] and *Paratypothorax andressorum* [32], and its boundary is marked by a fine continuous crest. This fossa is bounded anteriorly by the descending process of the nasal and the maxilla, dorsally by the ventral region of the nasal, ventrally by the maxilla and posteriorly by the lacrimal. The ventral margin of the maxilla is slightly convex and bears five foramina along its anterior portion, whereas along its mid-posterior portion it is marked by a longitudinal crest that borders the posteroventral end of the antorbital fossa. The anterior process is dorsoventrally tall, reducing its height anteriorly and ending up in a labiolingually depressed tip at the midpoint of the external naris. The dorsal surface of this process contacts the ventral surface of the posterior process of the premaxilla. The ascending process is short, contacting dorsally and anteriorly the nasal, and posteriorly the lacrimal. This process also forms the anterior portion of the antorbital fenestra. The posterior process is elongate and somewhat rectangular at its posterior end. It forms the entire ventral margin of the antorbital fenestra and forms the posterior end of this fenestra where it contacts the ventral process of the lacrimal. The antorbital fenestra is triangular in lateral view as in *Stagonolepis robertsoni* [7] and *S*. *olenkae* [59], although proportionally longer. The anterior margin of the antorbital fenestra is also oval as in these latter species, contrasting with the round condition present in *Aetosaurus ferratus* (SMNS 5770 S-16), *Stenomyti huangae* [18], *Paratypothorax andressorum* (SMNS 19003) and *Desmatosuchus* [42, 57].

#### Nasal

The nasal is a slim but long bone that forms the dorsal margin of the external naris. It tapers anteriorly until the contact with the anterior process of the premaxilla (Figs 2–4). There is a ventral projection on its middle portion, which forms the posterior margin of the external naris, and contacts the posterior process of the premaxilla as in most aetosaurs. In dorsal view, the nasal becomes wider posteriorly, contacting the maxilla and the lacrimal ventrally, and posteriorly the prefrontal and the frontal. In dorsal view, a triangular depression is observed, which starts at the midpoint of the nasal, on its midline. This feature continues posteriorly, diverging to the lateral sides, extending through the middle portion of the prefrontal, ending parallel to the posterior margin of the antorbital fenestra.

#### Prefrontal

The prefrontal is a triradiate element, with no ridges (Figs 2–4). Posteriorly it forms the upper anterior margin of the orbital fenestra. The dorsal process extends to form a small portion of the anterior margin of the orbital fenestra, also contacting the ventral margin of the frontal. The ventral process is short and, descending, contacts the upper posterior margin of the lacrimal. The anterior process extends to contact a small portion of the posterior portion of the nasal, and laterally the dorsal margin of the lacrimal. Parallel to the dorsal margin of the lacrimal, as mentioned above, the depression that started at the midpoint of the nasal continues, terminating before the ventral process of the prefrontal starts.

#### Frontal

The frontal is a rectangular bone, longer than wide, as in other aetosaurs. The frontal is two-thirds the length of the nasal,is poorly ornamented (as only three or four grooves are seen per cm), forming most of the superior portion of the orbit and is unfused (Figs 2–4). A depression in this bone surrounds the orbit. It also tapers anteriorly towards the nasal, forming a Z-shaped suture, laterally with the anterior tip of the prefrontal, and posteriorly with the parietal. The right frontal is broken at its anterior portion, overlapping the left frontal and nasal elements. Near the suture between this bone and the nasal, the continuation of the triangular depression that started at the midline of the nasal is seen, fitting the anterior tip of the frontal into this depression.

#### Postfrontal

The postfrontal is a triradiate bone, like the prefrontal. It is only preserved on the right side of the skull, where it forms, posterodorsally, a tiny portion of the orbit. It tapers posterodorsally to the ventral margin of the parietal, and anterodorsally to the posteroventral margin of the frontal.

#### Parietal

The parietals are unfused and broken transversally at their posterior portion, showing a semi-circular convex and thick shape, not flat, in occipital view. In this view, it is possible to see the posterior region of the endocranial cavity, completely displaced below the parietals and towards the right side. In dorsal view (Fig 4), it is possible to measure its width, although not at the midpoint, which is wider than the frontal (see Table 1). The parietal is more ornamented than the frontals, with three crests on the left parietal, and a few grooves (Fig 4).

#### Lacrimal

The lacrimal is an extensive three-pronged bone that forms most of the margin of the antorbital fenestra. Its posterior end forms the anterior margin of the orbit, dorsally tapering to the ventral margin of the prefrontal (Figs 2 and 3). It also tapers dorsally to the very anterior ventral portion of the frontal and the very posterior ventral portion of the nasal. Its ventral branch forms the posterior end of the antorbital fenestra, contacting the tip of the posterior process of the maxilla. The ventral branch also has a ridge along its length, near the suture with the prefrontal, which forms the posterior margin of the antorbital fossa. The anterior branch tapers to the dorsal margin of the antorbital fenestra, reaching the posterior margin of the ascending process of the maxilla. The ridge along the ventral branch continues on the anterior branch, following the suture with the ventral margin of the prefrontal and the nasal, forming the dorsal margin of the antorbital fossa.

#### Jugal

Excluded from the margin of the antorbital fenestra and fossa by the contact of the lacrimal with the maxilla, the jugal forms the ventral margin of the orbit (Figs 2 and 3). It antero-dorsally contacts a tiny portion of the lacrimal and anteriorly the posterior end of the maxilla. Despite the posterior portion of jugal being broken, the ridge that is present along the lateral surface of the maxilla continues along the jugal, and it is not dorsoventrally constricted. There is no ventral inclination of the jugal that is present in *Desmatosuchus* [42].

#### Quadrate

**Fig 5:**
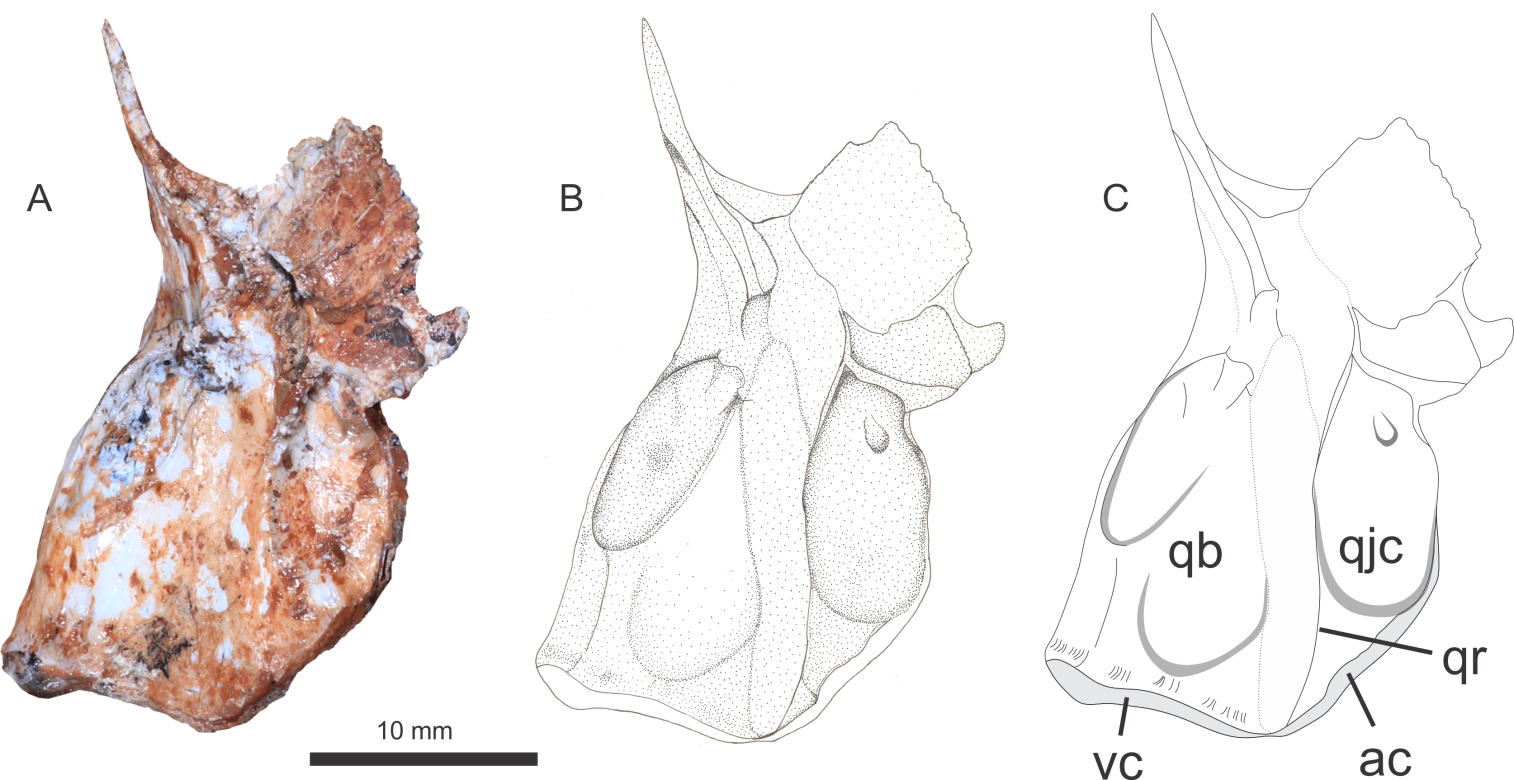
Right quadrate in dorsal-lateral view. Abbreviations: qb, quadrate body; qjc, quadratejugal contact; vc, ventral condyle; ac, anterior condyle; qr, quadrate ridge.

Only the right quadrate is preserved (Fig 5). In spite of being partially displaced, the quadrate is still articulated with the corresponding hemimandible. The quadrate body is bell-shaped in posterior view, and bears no projections, whereas the quadrate head is missing. The mandibular articulation has two condyles, one ventrally and the other anteriorly oriented, bounded by a thin ridge along the quadrate body and perpendicular to the ventral condyle. Moreover, a concave surface for the quadratojugal contact is present between the body ridge and the anterior condyle. It is not possible to observe either the synovial basal, the optical joints or the quadrate foramen.

#### Dentary

The dentary is gently convex along its ventral margin and posterodorsally it participates in the anterior margin of the oval external mandibular fenestra, and branches off at its posterior end (Fig 2). The upper branch contacts the surangular, forming the anterodorsal margin of the mandibular fenestra; the lower branch contacts the angular, forming the anterior and part of the ventral margins of the external mandibular fenestra, where a slight inflexion of the splenial on the ventral margin occurs, as in *Aetosaurus ferratus* [58]. The anterior portion of the dentary is edentulous, and only two teeth are preserved posteriorly in this region. At least two alveoli are present, and both are located alongside the preserved teeth.

#### Angular

The angular extends anteriorly forming the ventral margin of the mandibular fenestra (Fig 2). It is a narrow bone and slightly bowed on its ventral margin, maintaining its width throughout its length. On its posterior third, the angular contacts the ventral margin of the surangular, thickening and terminating in a small projection under the surangular.

#### Surangular

The surangular frames the mandibular fenestra dorsally and posteriorly. A rounded tuber on the dorsal margin of this bone is present (Fig 2), as in both *Stagonolepis* species, although it is shorter, as in *Stenomyti* [18]. The surangular sutures with the angular along its ventral margin on its posterior end, as in *A*. *ferratus*, in an oblique suture, and forms a posteroventral projection that expands dorsoventrally, where it houses a surangular foramen close to the glenoid fossa.

#### Splenial

The splenial is a thin and anteriorly sharp bone, observed in left medial view on the right side of the lower jaw. The displaced left lower jaw covers its posterior portion, and it is only possible to affirm that the splenial covers its medial surface. In right lateral view, the medial ventral margin of the splenial is visible right below the suture between the angular and the surangular, and it is visible only in this region (Fig 2). In ventral view, the splenial follows the dentary anteriorly in length, having a straight suture with it. Because the medial anterior tip of the splenial is broken, the mylohyoid foramen is not observable.

### Dentition

UFSM 11505 preserves homodont posteriorly recurved teeth with thecodont implantation, and with crown measuring 5–6 mm tall. Most teeth are preserved and were found *in situ*, with one tooth found isolated near the skull, which is most likely to be a dentary tooth based on its morphology. None of the teeth has constriction or swelling at the base of the crown, and all are distally recurved. The presence of serrations and the cross-section form vary depending on the position of tooth.

#### Premaxillary teeth

The right premaxilla bears five elongate teeth mid-posteriorly located, slightly recurved, without any constrictions and with the same width from crown base to apex (cylindrical-shaped). There is no evidence of wear facets, serrations or ornamentation. No teeth of the left premaxilla are preserved.

#### Maxillary teeth

**Fig 6:**
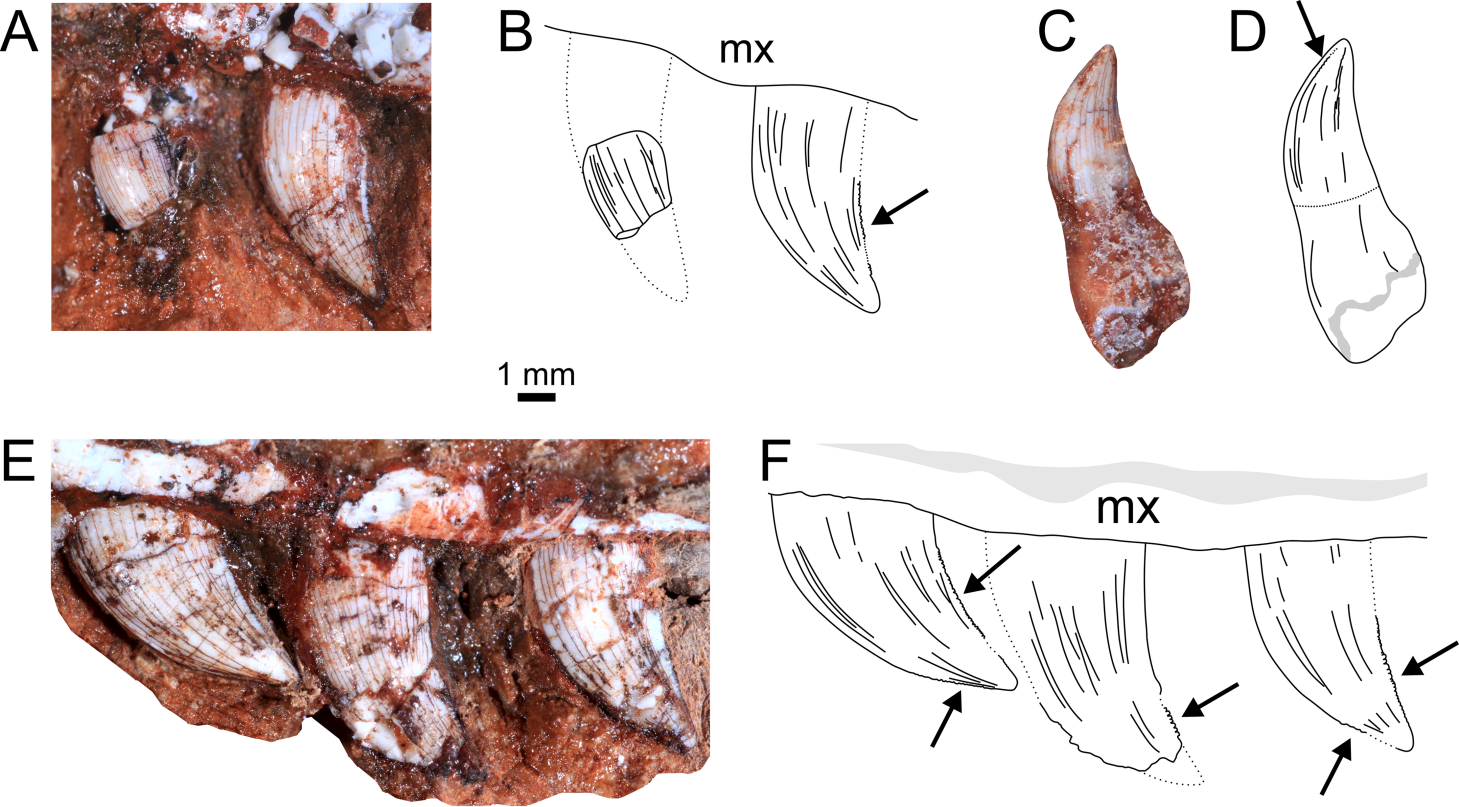
Teeth of referred specimen of *Aetosauroides scagliai* (UFSM 11505). (A-B) First two preserved teeth of the left maxilla. (C-D) Isolated dentary tooth. (E-F) Last three preserved teeth of the left maxilla. Arrows indicate serrations. Abbreviations: as Fig 2.

The maxillary teeth are labiolingually compressed, distally recurved with an oval cross-section. In lateral view, the maxillary tooth crowns possess similar edge morphology, being convex on the mesial margin and concave on the distal margin. There is a straight and serrated carina along the mesial and distal crown margins, with 8 denticles per mm (Fig 6). There are no fluting or ridges along the crown.

#### Dentary teeth

The dentary teeth are lanceolate in cross-section, with a pronounced distal carina along the crown. The crown is strongly distally recurved from its middle to apex, forming a “bent knee” at the base-apex midline. The teeth are smooth, without grooves or ridges, and are smaller than the maxillary teeth.

**Fig 7:**
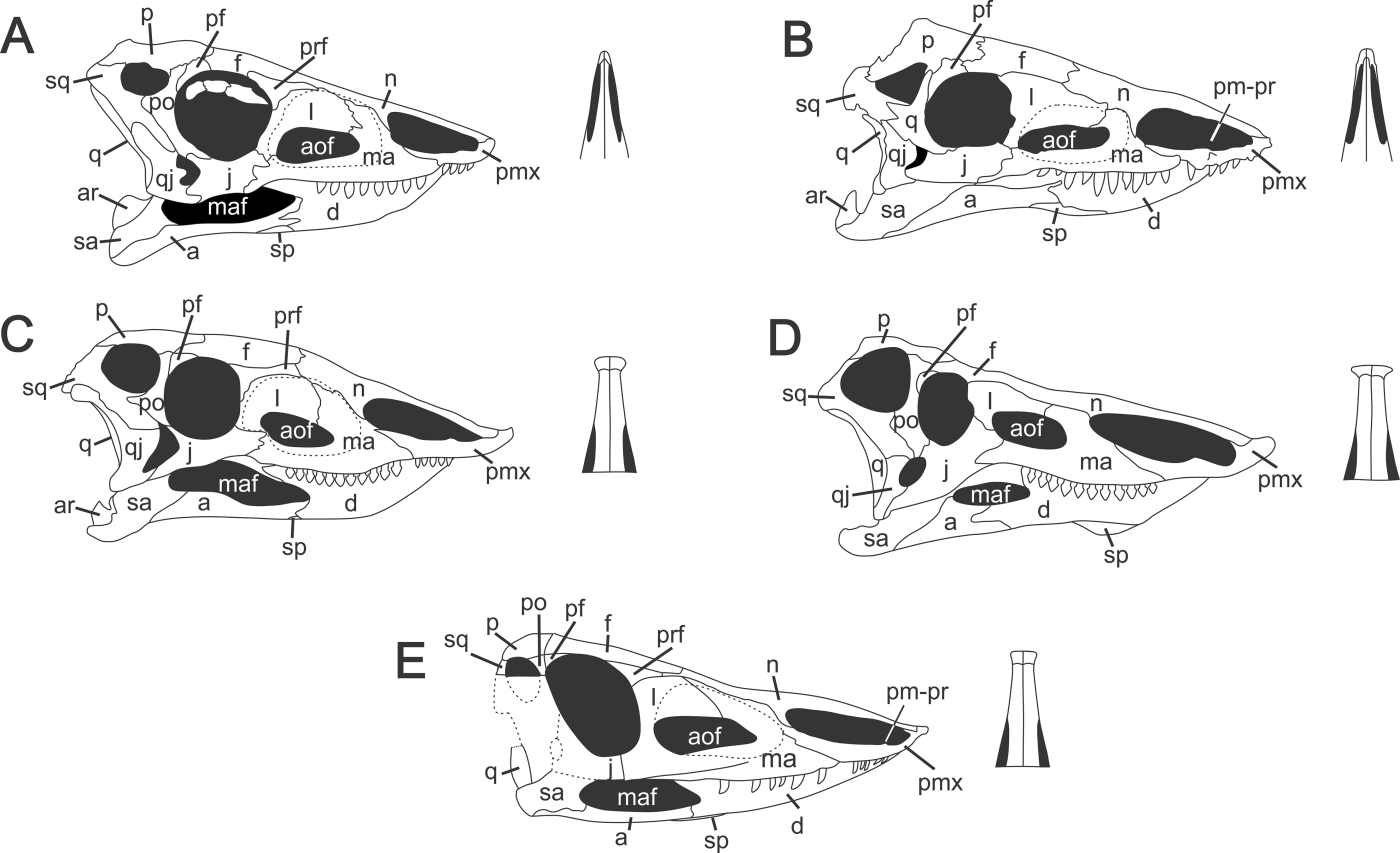
Skull reconstructions of some aetosaur species and, in detail, the referred snout in dorsal view of each species. (A) *Aetosaurus ferratus* (modified from Schoch [58]). (B) *Paratypothorax andressorum* (modified from Schoch & Desojo [32]). (C) *Stagonolepis robertsoni* (modified from Walker [7]). (D) *Desmatosuchus smalli* (modified from Small [42]). (E) *Aetosauroides scagliai* (modified from Desojo & Ezcurra [24]). Dotted lines represent unpreserved bones. Abbreviations: a, angular; aof, antorbital fenestra; ar, articular; d, dentary; f, frontal; j, jugal; l, lacrimal; m, maxilla; maf, mandibular fenestra; n, nasal; p, parietal; pf, postfrontal; pmx, premaxilla; pm-pr, dorsal projection of premaxilla; po, postorbital; prf, prefrontal; q, quadrate; qj, quadratojugal; sp, splenial; sq, squamosal.

The amount of new information allowed us to reconstruct a model of the skull of *Aetosauroides scagliai*, based on the models of Casamiquela [15, 28] and Desojo & Ezcurra [24]. This reconstruction is shown in Fig 7 along with reconstructions of *Aetosaurus ferratus* [58], *Paratypothorax andressorum* [32], *Stagonolepis robertsoni* [7] and *Desmatosuchus smalli* [42, 57].

## Phylogenetic analysis

The present description of the skull in UFSM 11505 has provided a set of previously unknown cranial traits of *Aetosauroides scagliai* (e.g. premaxilla, tooth morphology; including new information on eight characters of the data matrix) and on aetosaur skull anatomy in general. These features provide the necessary information to update existing cladistic analyses and, therefore, answer some questions about aetosaur phylogeny (see discussion).

For our phylogenetic analysis, we use the most recent data matrix [60], which was originally composed of 83 characters and 28 taxa. We combine the scorings of the specimen SMSN 19003, kept by Parker [60] as a separate OTU (Operational Taxonomic Unit), with *Paratypothorax andressorum* following the assignment made by Schoch & Desojo [32]. Thus the resulting matrix contains 83 characters and 28 taxa by the addition of UFSM 11505 as a separate OTU to test the specimen position.

Parker [60] ordered seven characters, and we kept the same characters as additive (ordered). The rauisuchid *Postosuchus kirkpatricki* Chatterjee [61] was used to root the recovered most parsimonious trees (MPTs) and *Revueltosaurus callenderi* [62], a pseudosuchian, was used as a second outgroup, as *R*. *callenderi* is consistently found as the sister-group of Aetosauria [63].

The data matrix was analyzed under equally weighted maximum parsimony using TNT 1.5 [64]. Zero-length branches among any of the recovered MPTs were collapsed according to rule 1 of Coddington & Scharff [65].

Five previously unknown characters for *A*. *scagliai* were scored for UFSM 11505 (1:1; 12:0; 13:0; 31:1, 35:0) and four characters were modified from the previous matrix for the holotype (5:0→1, 19:1→0, 30:0→1, 35:0→?). For more details, see Discussion.

**Fig 8:**
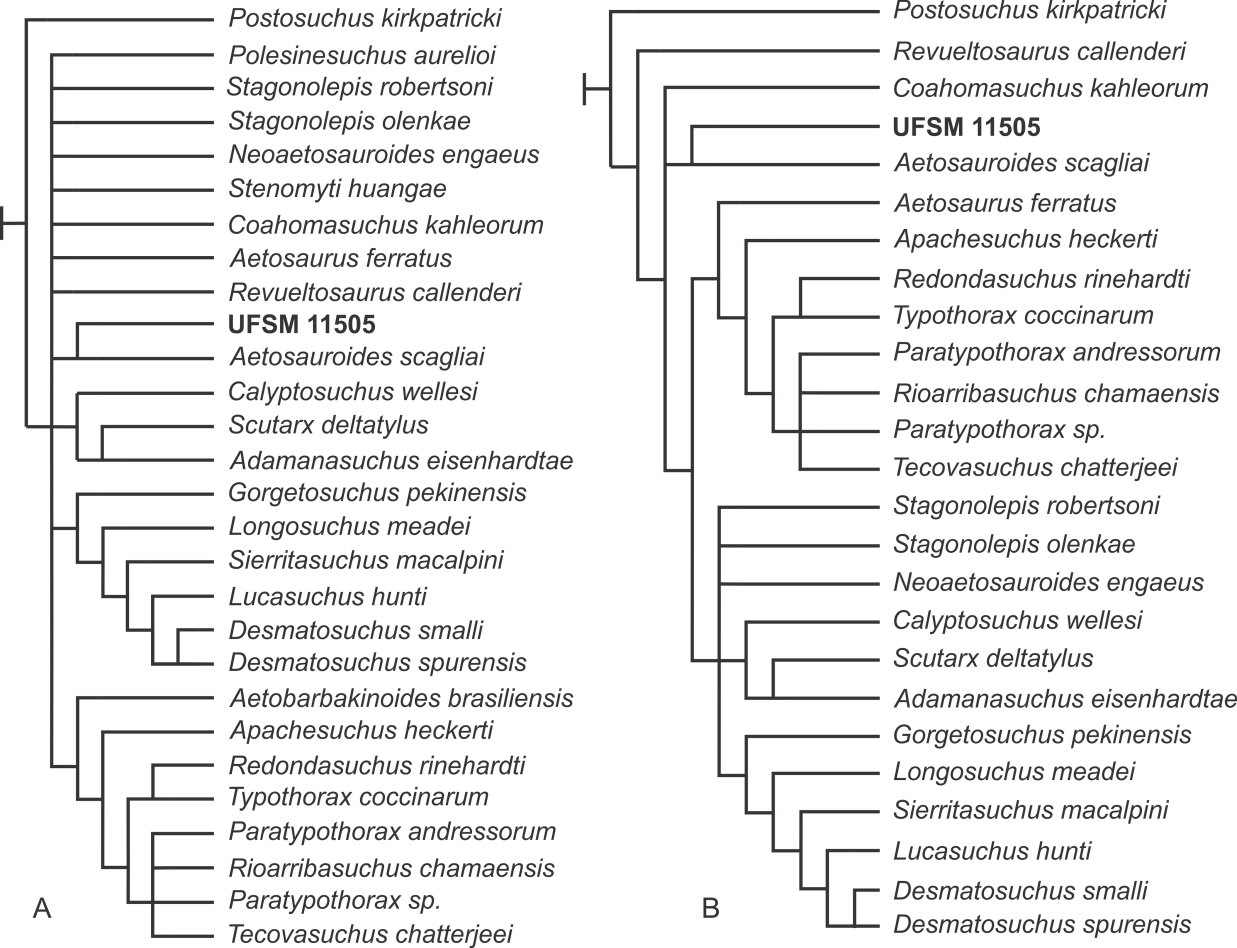
**Strict (= Nelsen) consensus of 34 (A) and 6 (B) MPTs.** (A) Strict consensus of the 34 MPTs with large polytomy. (B) Strict consensus of the 6 MPTs after *a priori* pruning of *Aetobarbakinoides brasiliensis*, *Stenomyti huangae* and *Polesinesuchus aurelioi*.

**Fig 9:**
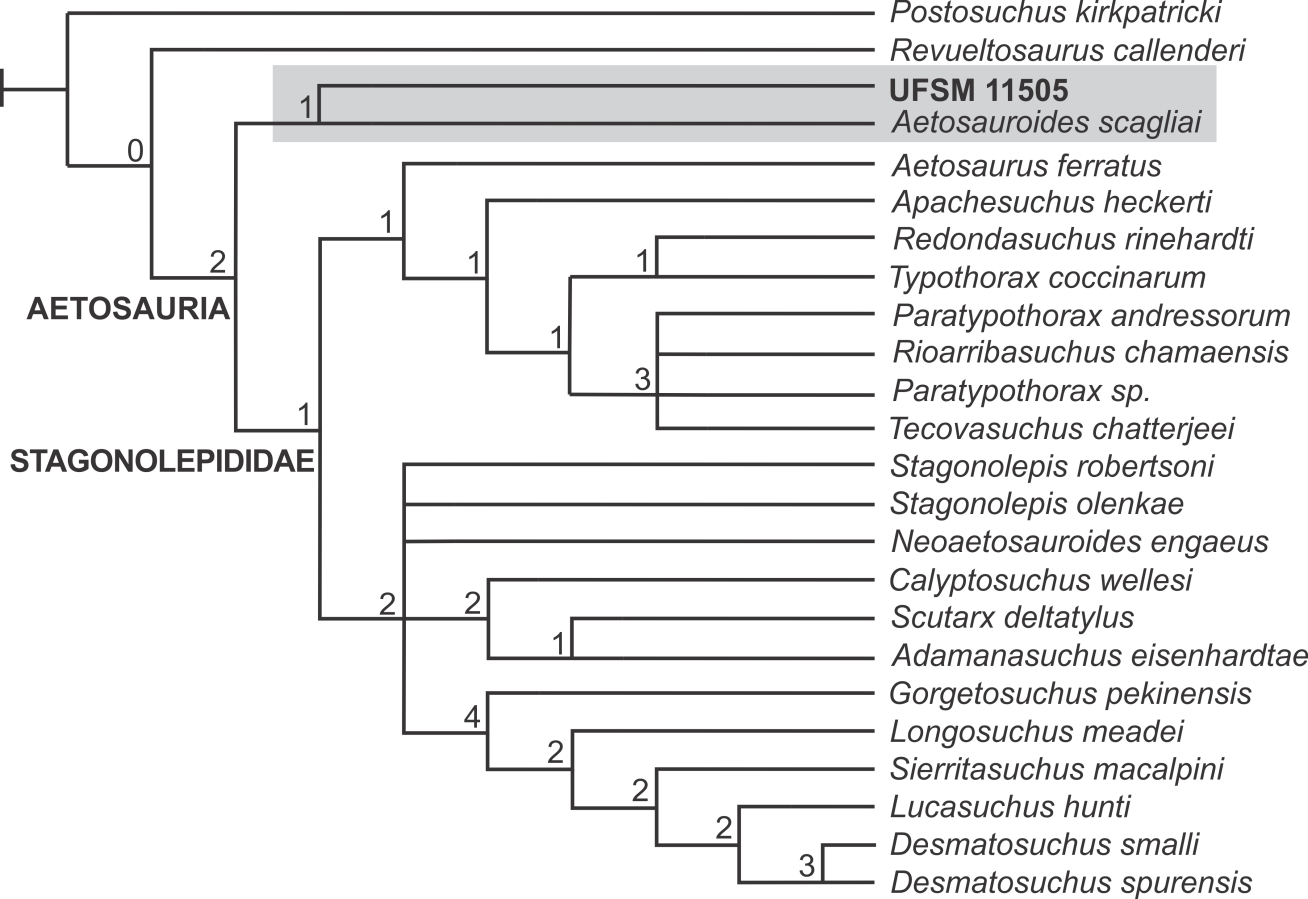
Strict (= Nelsen) consensus of the 6 MPTs used for this study after *a posteriori* pruning of *Coahomasuchus kahleorum*. Bremer support values are shown for all nodes.

We ran our analysis of the adapted matrix from Parker [60] using traditional search of 100 replications of Wagner trees (with random addition sequence), followed by TBR branch swapping algorithm (holding 10 trees per replicate). The analysis resulted in 34 MPTs with 205 steps. The strict consensus of this tree is provided in Fig 8A, featuring a large polytomy at the base of Aetosauria. To resolve this polytomy, we ran positional congruence (reduce) index (PCR) according to methodology presented in Pol & Escapa [66], aiming to measure the stability of used taxa. This analysis recovered *Aetobarbakinoides brasiliensis* [22], *Stenomyti huangae* [18], and *Polesinesuchus aurelioi* [23] as the most unstable taxa in the analysis. We excluded these three taxa *a posteriori*, resulting in a matrix of 83 characters and 25 taxa, and ran an analysis with the same configuration as the first analysis. This analysis resulted in 6 MPTs with 193 steps. The strict (= Nelsen) consensus tree is showed in Fig 8B. As *Coahomasuchus kahleorum* [17] formed a trichotomy with both the unnamed group formed by UFSM 11505 + *A*. *scagliai* and the Stagonolepididae (*sensu* Parker [60]), and an examination of the 6 MPTs demonstrated that this taxon occurs in 2 possible positions, it was pruned *a posteriori*. The strict (= Nelsen) consensus tree of these 6 MPTs with Bremer support after pruning of *Coahomasuchus kahleorum* is provided (Fig 9).

## Discussion

Both the osteology description and phylogenetic analysis revealed some interesting traits for *Aetosauroides scagliai*. Here we discuss the new character states recognized for this taxon.

Teeth have a variable morphology within Aetosauria. Taxa with plesiomorphic character states [60], such as *Aetosaurus ferratus*, Norian age, presents bulbous and not recurved crowns [58], as in *Stenomyti huangae* [18] and *Paratypothorax andressorum* [32]. None of them have serrated teeth. This contrasts with tooth morphology of *Stagonolepis robertsoni* [7], of Carnian age, which has a “leaf-like” shape in labial/lingual view, mediolaterally compressed and with a denticulate mesial margin [7]. UFSM 11505 teeth, as mentioned in the description, are oval in occlusal view and posteriorly recurved in labial/lingual view, without a constriction between root and crown, and possess denticles on both the mesial and distal regions. In Parker contribution [60], tooth morphology is described for *A*. *scagliai*, because PVL 2059, its referred material, has one maxillay tooth and poorly preserved dentary teeth. Nonetheless, it is not possible to know if the only maxillary tooth of PVL 2059 is recurved or not, as its crown is broken. Accordingly, we modified the state of the character 35 of Parker [60] for *A*. *scagliai* from fully recurved (state 0) to unknown (?). Within Archosauriformes, tooth serrations are present in nearly all in-group clades and considered a plesiomorphic condition, as in *Postosuchus kirkpatricki* and *Revueltosaurus callenderi* [39, 67], used here as outgroup and second outgroup, respectively. As *A*. *scagliai* is herein recovered as the sister-taxon of all other members of Aetosauria, confirming previous studies [22, 32, 60, 68], and both UFSM 11505 and PVL 2059 having serrations on their teeth, these could indicate the presence of denticles as a plesiomorphic characteristic within Aetosauria.

The variation of morphology between premaxillary and maxillary teeth is also another feature present in UFSM 11505. As mentioned before, premaxillary teeth are cylindrical in shape, and slightly recurved, in contrast with the oval shape in occlusal view and recurved maxillary teeth. Variation between premaxillary and maxillary teeth is also seen in *Aetosaurus*, in which maxillary teeth are “more bulbous and slightly longer than those of the premaxilla” [58].

Another peculiarity within Aetosauria is the shape of premaxilla, which is one of the key elements to understand [Aetosauria phylogeny more completely. Two general shapes of the premaxilla in dorsal view are known for aetosaurs: an anteromedially tapering and a laterally expanded premaxilla, known as “shovel-shaped” [60, 69]. *Desmatosuchus smalli* [42, 57] and *Stagonolepis robertsoni* [7] both posses a laterally expanded premaxilla, maintaining a nearly constant width until the apex [60]. In *Aetosaurus ferratus* [58], *Stenomyti huangae* [18] and *Paratypothorax andressorum* [32], the premaxilla tapers anteromedially. Neither the holotype of *A*. *scagliai*, PVL 2073, and referred materials in previous studies [15, 28, 29, 38, 60] had the premaxilla preserved, so it was not possible to tell which shape it had. However, UFSM 11505 preserves a premaxilla that is gently laterally expanded, herein described as having a “smooth shovel-shape”, more similar to *Desmatosuchus* than to *Aetosaurus*. In the phylogeny proposed by Parker [60] and herein corroborated, *Aetosauroides scagliai* is a non-stagonolopididae aetosaur sister with all Stagonolepididae aetosaurs. Two branches form Stagonolepididae: Desmatosuchia and Aetosaurinae. *Desmatosuchus* and *Stagonolepis* are branched within Desmatosuchia, and *A*. *ferratus*, *S*. *huangae* and *P*. *andressorum* are branched within Aetosaurinae. As *Aetosauroides scagliai* is a basal taxon within Aetosauria, the character presented by UFSM 11505 supports the plesiomorphic condition for the clade, and could indicate that the expansion of the premaxilla may have a tendency to disappear in Aetosaurinae, as in *Aetosaurus*, *Stenomyti* and *Paratypothorax*, or expand more, as defined by Parker [60] in Desmatosuchia, as in *Stagonolepis* and *Desmatosuchus*.

The premaxilla also has, in some taxa, its dorsal surface with a prominent dorsal tubercle that extends into the external naris. This feature was not described for *A*. *scagliai* (PVL 2059) because the exact portion where this tubercle is located is broken, and therefore the tubercle is not preserved. However, it is possible to observe a small tubercle on UFSM 11505, less prominent than that of *Stagonolepis* and *Desmatosuchus*, and similar to that of *Stenomyti*.

The jugal, according to Nesbitt [67], in most archosauriforms, its ventral margin is horizontally oriented. This is also seen in *Paratypothorax andressorum*, and is in contrast with other aetosaurs, such as *Desmatosuchus*, *Stagonolepis robertsoni* and *Neoaetosauroides engaeus* Bonaparte [16], which have a posteroventrally inclined ventral margin of the jugal. The jugal was not preserved either in the holotype of *A*. *scagliai* or in the referred materials [15, 28, 29, 38, 60]. In lateral view, the jugal of UFSM 11505 appears to have a nearly horizontal ventral margin, with no inclination in any direction. Additionally, the anterior process of the jugal is excluded from the border of the antorbital fenestra by the contact between the lacrimal and maxilla in UFSM 11505, as seen in other pseudosuchians such as *Postosuchus kirkpatricki* and *Revueltosaurus* [67], both used as outgroups. The jugal is also excluded from the antorbital fenestra in *Stenomyti*, *Aetosaurus*, *Paratypothorax* and *Coahomasuchus kahleorum* [17], contrasting with the character state present in Desmatosuchia (*sensu* Parker [60]), where the jugal participates in the formation of the antorbital fenestra margin, as in *Desmatosuchus* and *Stagonolepis*. Therefore, *A*. *scagliai* retains basal plesiomorphic character states of the jugal.

UFSM 11505 presents, on the dorsal margin of the surangular, a prominent rounded tuber (state 1 of character 5). This character state is seen in both *Stagonolepis* species, *S*. *olenkae* Sulej [59], *S*. *robertsoni* and also in *Stenomyti huangae*. As the posterior portion of the mandible was previously unknown for *A*. *scagliai*, the prominent rounded tuber was here assigned as present (state 1) for this species.

Regarding the parietals and frontals, Parker [60], in his data matrix, assigned to character 19 (comparison of transverse width between frontals and parietals at anteroposterior mid-points) a frontal element wider than the parietal (state 1), as in *Desmatosuchus* and *Stagonolepis*, without a description or comment on the characters list. On the first descriptions of *Aetosauroides* skull [15, 28, 29], no observations of the size of these elements were taken, as the parietals were not preserved. On UFSM 11505, although the parietals are broken at their posterior portion, it is possible to verify that their widths at the anterior portion are wider than the midpoint of frontals (see Table 1). Hence, it was assigned here as parietals wider than the frontals (state 0), as occur in *Aetosaurus ferratus* and *Stenomyti*.

On the “slipper-shaped” mandible of aetosaurs, Parker [60] modified a character on the data matrix of Heckert & Lucas [17] (character 15) in two different characters (29 and 30 of Parker [60]). Character 29 was described as the ventral margin of the mandibular ramus in lateral view, which is gradually convex in *A*. *scagliai* (state 0), an autapomorphy for this species [24], and with an inflexion of the splenial or the dentary in other Stagonolepidid (*sensu* Parker [60]) aetosaurs. Character 30 was described as the anterior end of the dentary in lateral view, which, as described by Parker [60], has a rounded termination in *A*. *scagliai*, as the author observed in PVL 2059, and tapers to an acute point in all other aetosaurs, as contributing to the “slipper-shape” of the mandibular ramus. However, this character is not preserved in PVL 2059, and it is not possible to tell if the anterior end is indeed rounded, contradicting the affirmation. In UFSM 11505, the anterior end tapers to an acute point (state 1), as all other Aetosauria taxa. However, it does not contribute to the “slipper-shaped” mandible, as *A*. *scagliai* has a gradually convex mandibular ramus. Therefore, the state of character 30 was modified from state 0 (dentary with rounded anterior end) to 1 (anterior end of dentary tapers to an acute point) for *A*. *scagliai*.

We also discuss some specific questions regarding the new states of characters. Here we summarize and discuss briefly two questions we found relevant to be answered in this study.

1) Is the exclusion of the maxilla from the external naris a case of intraspecific variation?

In archosaurs, the skull exhibits a trend to exclude the maxilla from the formation of the external naris, and it is preserved to a variable degree in the descendent groups [70], such as in Pseudosuchia where the maxilla participates in the external naris margin [67]. The character state of the maxilla excluded from the external naris is present in *A*. *scagliai* and the outgroup *Postosuchus kirkpatricki* but not in all other Aetosauria. In both referred materials of *A*. *scagliai* in Desojo & Báez [26] and Parker [60] (PVL 2059 and PVL 2052), this autapomorphy is preserved. Considering the trend in archosaurs to exclude the maxilla from the external naris and the place occupied by UFSM 11505 in this phylogeny as an early diverging aetosaur taxon, in a dichotomy with *A*. *scagliai*, confirms this condition as a plesiomorphic state of this character. On the other hand, in Stagonolepidid aetosaurs, this character is modified until the maxilla participates in the margin of the external naris [7, 24, 32, 58, 59].

Additionally, in UFSM 11505, the descending process of nasal and ascending process of premaxilla are covering the maxilla and fractured in their tips, which makes the maxilla appears to be participating of the external naris margin. Thereby, this could be an intraspecific variation of this character, implying the tendency in Aetosauria of the participation of the maxilla in the external naris margin.

2) The smooth shovel-shaped premaxilla: are this and other characters states in *A*. *scagliai* plesiomorphic?

To understand the position occupied by *Aetosauroides*, first it is necessary to understand the problem of the phylogenetic placement of *Aetosaurus ferratus* within Aetosauria.

In a revision of *Aetosaurus ferratus*, Schoch [58] recognized for this taxon a shorter premaxilla with an edentulous and very short anterior portion, and the anterior end of premaxilla without lateral expansion. The author defined them as plesiomorphic characters states in accordance to other phylogenetic studies that placed *Aetosaurus* at the base of Aetosauria (e.g. [17, 69]), and *Aetosauroides* as a sister taxon of *Stagonolepis* [69]. However, recent phylogeny studies recovered not only *A*. *ferratus* nested within Stagonolepididae [19] but also in a polytomy with *Coahomasuchus kahleorum* and Typothoracinae [60], in contrast with studies mentioned above and others that pulled *Aetosaurus* to the base of the tree, along with *Aetosauroides scagliai* [22, 24, 68].

It is considered here that the *Aetosaurus ferratus* characters mentioned above as plesiomorphic are unique within Aetosauria. For Cerda & Desojo [71] and Schoch & Desojo [32], these traits regarded as plesiomorphic are more likely to be interpreted as of a juvenile form. Notwithstanding, *Aetosaurus ferratus* presents another set of characters that seem to show trends among derived taxa within the group. In this regard, *Aetosauroides scagliai* possesses features that resemble both with characters of the outgroup and with early characters of Aetosauria, independent of ontogenetic states: (1) the exclusion of the maxilla from the margin of the external naris, present in *Aetosauroides* but not in *Aetosaurus* nor in any other Aetosauria taxon; (2) smoothly expanded premaxilla may indicate a state of character in which the expansion could disappear, as in *Aetosaurus* and *Stenomyti*, or expand more, as in *Stagonolepis* and *Desmatosuchus*; (3) the presence of conical teeth, not bulbous, is a characteristic within Pseudosuchia [36] and present in *Aetosauroides*, but not in other Aetosauria taxa, which bear bulbous teeth (*Aetosaurus*, *Stagonolepis*, *Stenomyti*, *Desmatosuchus*); (4) the presence of premaxillary teeth is a “primitive” characteristic in tetrapods [70] and is present in *Aetosauroides* and Typothoracinae (*sensu* Parker [60]), but absent in derived taxa as *Desmatosuchus*.

As the herein presented phylogenetic analysis strongly supports *Aetosauroides scagliai* at the base of the tree, the state of characters mentioned above could be considered plesiomorphic for *Aetosauroides*, corroborating previous studies [22, 32, 60].

## Conclusions

The recognition of hitherto unknown skull character states for *Aetosauroides scagliai*, like the smooth shovel-shaped premaxilla, the recurved and unconstricted teeth, and the presence of a tuber on the surangular allows greater knowledge on the anatomy of this species and the identification of plesiomorphic characters states within Aetosauria. Additionally, the result of our phylogenetic analysis confirms *A*. *scagliai* at the base of Aetosauria and reinforces the former as the earlier species within this group.

Our analysis, along with other studies (e.g. [24, 32, 58, 60, 68, 72]) emphasizes the importance of detailed descriptions and phylogeny reviews to define the characters, aiming to recognize them as plesiomorphic or as characteristics of juveniles that may vary with ontogeny. Hence, the obtainment of a more accurate phylogeny and a better knowledge of Aetosauria in general can be achieved in future studies.

## Supporting information

S1 Supporting Information Character/Taxon matrix. (TXT)

## Abbreviations

MCP: Museu de Ciências e Tecnologia, Porto Alegre, Brazil
PVL: Paleontología de Vertebrados, Instituto “Miguel Lillo”, San Miguel de Tucumán, Argentina
SMNS: Staatliches Museum für Naturkunde, Stuttgart, Germany
UFRGS-PV: Universidade Federal do Rio Grande do Sul, Laboratório de Paleovertebrados do Instituto de Geociências, Porto Alegre, Brazil
UFSM: Universidade Federal de Santa Maria, Santa Maria, Brazil
